# Supplementary material for: Prevalence of overweight and obesity and their impact on academic performance and psychological well-being among university students in 2024 in Bangladesh
Source: PLoS One. 2024 Dec 10;19(12):e0315321. doi: 10.1371/journal.pone.0315321 (PMC11630606; doi:10.1371/journal.pone.0315321)
Supplement: S2 File — (PDF) [file pone.0315321.s002.pdf]

Questionnaire on

Prevalence of Overweight and Obesity and Their Impact on Academic Performance and Psychological Well-being among Students of Jahangirnagar University

Dear Participants,

We are members of the Jahangirnagar University Research Society and want to conduct a survey on obesity for academic research. The main objective of this survey is to know the prevalence of overweight and obesity among Jahangirnagar University students and its impact on academic performance and mental well-being. We assure you that no personal information (such as name, mobile number, address, etc.) will be asked for anywhere in the survey and your information will be kept confidential. Results will produced anonymously. You can stop or withdraw your participation at any point in the survey. It will take about 15-20 minutes to complete this survey. We appreciate your participation and hopefully, your valuable information will help us in this survey.

Do you freely and voluntarily agree to take part in this study?

1. Yes 2. No

If Yes, continue, and if No then Stop.

Section A: Socio-Demographic Information			
**Serial**	**Options**		**Response**
A1	Age (year)		
A2	Gender	1 = Male<br>2 = Female	
A3	Height (Feet and Inches)		
A4	Weight (kg)		
A5	Faculty	1 = Faculty of Mathematical, Physics and Biology<br>2 = Faculty of Social Science<br>3 = Faculty of Arts and Humanities<br>4 = Faculty of Business Studies	

A6	Academic Year (Year)	1 = Second Year<br>2 = Third Year<br>3 = Fourth Year<br>4 = Master's	
A7	Monthly Family Income	1 = Middle Class<br>2 = Lower-Middle Class<br>3 = Upper-Middle Class<br>4 = Higher Class	
A8	Residential status	1 = Residential Hall<br>2 = Home<br>3 = Match	
A9	History of family obesity	1 =Present<br>2= Absent	

Section B: Academic Performance Measurement		
B1	CGPA	

Section C: Rosenberg Self-Esteem Scale					
		**Strongly Disagree**	**Disagree**	**Agree**	**Strongly agree**
C1	At times I think I am no good at all				
C2	I feel that I have a number of good qualities				
C3	All in all, I am inclined to think that I am a failure				
C4	I am able to do things as well as most other people				
C5	I feel I do not have much to be proud of				
C6	I take a positive attitude toward myself				
C7	On the whole, I am satisfied with myself				
C8	I wish I could have more respect for myself				
C9	I certainly feel useless at times				
C10	I feel that I'm a person of worth				

Section D: Dietary variables			
		**Yes**	**No**
D1	Eat red meat once a day		
D2	Try to eat fiber food		
D3	Avoid fat and cholesterol foods		

D4	Eat fruits and vegetables (<5 times/day)		
D5	Avoid breakfast		
D6	How many times a day do you eat?		
D7	How often do you have breakfast?		

Section E: International Physical Activity Questionnaire (IPAQ-7)		
		**Response**
**E1**	In the last 7 days, how many days did you do vigorous physical activity such as heavy lifting, digging, or fast cycling?	1. ________day/week)<br>2. No vigorous physical activity<br>(If the answer to question 1 is 2, then directly go to question D3)
**E2**	How much time do you usually spend doing vigorous physical activity on one of those days?	1.________hour/day<br>2.________minute/day<br>3. Don't know/Not sure
**E3**	In the past 7 days, how many days did you do moderate physical activity such as carrying light weights, cycling at a steady pace, or playing doubles tennis?<br>Don't include walking.	1. ________week/day<br>2. No moderate physical activity (go directly to question D5 if the answer to question 3 is 2)
**E4**	How much time do you usually spend doing moderate physical activity on one of those days?	1.________minute/Day<br>2. ________minute/Day<br>3. Don't know / Not sure
**E5**	In the last 7 days, how many days did you walk at least 10 minutes at a time?	1. ________day/week<br>2. Don't walk (If the answer to question no 5 is 2, I will go directly to question D7)
**E6**	How long do you usually walk on one of those days?	1. ________hour/day<br>2. ________minute/day<br>3. Don't know/Not sure
**E7**	In the last 7 days, how much time did you spend sitting?	1. ________hours/days<br>2. ________minutes/days<br>3. Don't/Not sure

Section F: Smoking and Drinking Status			
**F1**	Do you drink five or more drinks on any occasion?	1. Yes<br>2. No	
**F2**	Do you use one or more of the following tobacco products? (cigarettes, snuffs, weed, etc.)	1. Yes<br>2. No	

Thank You
