# Supplementary material for: Prevalence of overweight and obesity and their impact on academic performance and psychological well-being among university students in 2024 in Bangladesh
Source: PLoS One. 2024 Dec 10;19(12):e0315321. doi: 10.1371/journal.pone.0315321 (PMC11630606; doi:10.1371/journal.pone.0315321)
Supplement: Attachment — Submitted filename: review_obesity.docx [file pone.0315321.s004.docx]

My comments regarding this article (Prevalence of Overweight and Obesity and their Impact on Academic Performance and Psychological Well-being among University Students).

The title is good; however, it is better to write all the three elements (time, place and person) now only person can be found.

Suggestion Prevalence of Overweight and Obesity and their Impact on Academic Performance and Psychological Well-being among University Students in 2024, in name of the country.

Abstract: it is 294 words, please summarize the information as well as, AI detected 51%, change it and paraphrase the whole sentences.

Keywords are good.

Introduction: it is good written and structured. In the reference number 43 already they approved that overweight and obesity can impact negatively on mental health, why you still repeated same title, as well as tell the reader much more evidence to conduct the recent study, give the reasonable reason. Try to criticize the previous study.

The aim is clear.

Method section:

23% of the method section has been written by AI, please review it carefully and rewrite the sentences.

The authors wrote the study design and participants.

I recommend them to change the sub heading like (study setting, study design participants, questionnaire, and so on).

Then please clarify why you select students only in one university, give the reason?.

You reported that you used stratified sampling, same tell the reader why you select this method? And it is better to have and present the sampling technique through diagram.

By which software you selected 600 students? Is it the total sample size? Or the whole number of the students?

You didn’t mentioned the exclusion criteria and inclusion criteria. You didn’t mention the consent was written paper or taken orally, please describe it.

Please mention the *P* value.

Result section: 22% has been written by AI, please change and paraphrase it. The whole section is good structured and well written.

Discussion section: 42% has been written by AI. You didn’t mention the strength points of the study please describe your strong points and then mention the limitation, during mentioning the limitations you didn’t use scientific terms such as report bias. You used some other words please review the words. Also, tell the redear how this type of bias can be effective on your result?

Conclusion: 100% of discussion part has been by AI, please change the whole section and humanize it. And start with in conclusion or some other phrases which can be used for discussion part. Please don’t repeat the aim of the study inside conclusion part.

In addition, add recommendations and the future work in the same area, as well as, your advise for students and the whole community.

The reference list

The reference list has several issues, including incomplete citations, uneven formatting, missing DOIs or URLs, repetition, contradictory journal names and volume numbers, and inconsistent style. Some entries on the list lack DOIs or URLs, while others contain duplicate entries or cite comparable research more than once.

106 reference were used do you think all of them are necessary? Please revise them and use few of them.
