# Supplementary material for: Prevalence of overweight and obesity and their impact on academic performance and psychological well-being among university students in 2024 in Bangladesh
Source: PLoS One. 2024 Dec 10;19(12):e0315321. doi: 10.1371/journal.pone.0315321 (PMC11630606; doi:10.1371/journal.pone.0315321)
Supplement: Attachment — Submitted filename: Response to Reviewers.docx [file pone.0315321.s005.docx]

**Author responses to the review comments:**

We would like to express our sincere gratitude to the reviewers and the Editors for their valuable comments. We have considered all the comments made by the reviewers and thoroughly revised and formatted the manuscript accordingly. A detailed response to each of the comments is provided below:

| **Academic Editor Comments:** | **Response** | **Note** |
| --- | --- | --- |
| Thank you for submitting your manuscript to PLOS ONE. After careful consideration, we feel that it has merit but does not fully meet PLOS ONE’s publication criteria as it currently stands. Therefore, we invite you to submit a revised version of the manuscript that addresses the points raised during the review process.  Please include the following items when submitting your revised manuscript:  A rebuttal letter that responds to each point raised by the academic editor and reviewer(s). You should upload this letter as a separate file labeled 'Response to Reviewers'.  A marked-up copy of your manuscript that highlights changes made to the original version. You should upload this as a separate file labeled 'Revised Manuscript with Track Changes'.  An unmarked version of your revised paper without tracked changes. You should upload this as a separate file labeled 'Manuscript'. | Thank you very much for carefully checking the manuscript and providing insightful comments.  All required files are uploaded to the journal system. | Revised texts are in red color. |
| **Journal Requirements:** | **Response** | **Note** |
| 1. Please ensure that your manuscript meets PLOS ONE's style requirements, including those for file naming. The PLOS ONE style templates can be found at  https://journals.plos.org/plosone/s/file?id=wjVg/PLOSOne_formatting_sample_main_body.pdf and  https://journals.plos.org/plosone/s/file?id=ba62/PLOSOne_formatting_sample_title_authors_affiliations.pdf | Thanks. We revised the manuscript following the PLOS ONE style. | Revised texts are in red color.  Page: 1-20 |
| 2. In the ethics statement in the Methods, you have specified that verbal consent was obtained. Please provide additional details regarding how this consent was documented and witnessed, and state whether this was approved by the IRB | Thanks. We add it in the Methods section. Before starting the survey, the respondents were informed about the objective of the study and asked whether they wanted to participate in the study voluntarily or not. If they aged then the survey continued. | Revised texts are in red color.  Page: 6 |
| 3. You indicated that you had ethical approval for your study. In your Methods section, please ensure you have also stated whether you obtained consent from parents or guardians of the minors included in the study or whether the research ethics committee or IRB specifically waived the need for their consent. | Thanks. We received ethical approval from the IRB of Jahangirnagar University, Bangladesh. Additionally, we take consent from the participants because we value the privacy of the respondents as well. | Revised texts are in red color.  Page: 6 |
| 4. In this instance it seems there may be acceptable restrictions in place that prevent the public sharing of your minimal data. However, in line with our goal of ensuring long-term data availability to all interested researchers, PLOS’ Data Policy states that authors cannot be the sole named individuals responsible for ensuring data access (http://journals.plos.org/plosone/s/data-availability#loc-acceptable-data-sharing-methods).  Data requests to a non-author institutional point of contact, such as a data access or ethics committee, helps guarantee long term stability and availability of data. Providing interested researchers with a durable point of contact ensures data will be accessible even if an author changes email addresses, institutions, or becomes unavailable to answer requests.  Before we proceed with your manuscript, please also provide non-author contact information (phone/email/hyperlink) for a data access committee, ethics committee, or other institutional body to which data requests may be sent. If no institutional body is available to respond to requests for your minimal data, please consider if there any institutional representatives who did not collaborate in the study, and are not listed as authors on the manuscript, who would be able to hold the data and respond to external requests for data access? If so, please provide their contact information (i.e., email address). Please also provide details on how you will ensure persistent or long-term data storage and availability. | Thanks. We revised the data availability statement as “The data is attached as a supplementary file of this manuscript.” The data is attached as a supplementary file of the revised version of the manuscript. | Revised texts are in red color.  Page: 16 |
| **Reviewer 1 Comments:** | **Response** | **Note** |
| many thanks for sending this paper to review. i have reviewed and i wrote my comments separately in the different attached file. the topic is good and informative for the students and as well as for the general population. this topic is important since obesity can make different health issues inside community. | Thank you very much for carefully checking the manuscript and providing insightful comments. | Revised texts are in red color. |
| My comments regarding this article (Prevalence of Overweight and Obesity and their Impact on Academic Performance and Psychological Well-being among University Students).  The title is good; however, it is better to write all the three elements (time, place and person) now only person can be found.  Suggestion Prevalence of Overweight and Obesity and their Impact on Academic Performance and Psychological Well-being among University Students in 2024, in name of the country. | Thanks for your suggestion. We revise the title of the manuscript. | Revised texts are in red color.  Page: 1 |
| Abstract: it is 294 words, please summarize the information as well as, AI detected 51%, change it and paraphrase the whole sentences. | We appreciate your comments. We revise the Abstract. | Revised texts are in red color.  Page: 1-2 |
| Keywords are good. | Thanks. |  |
| Introduction: it is good written and structured. In the reference number 43 already they approved that overweight and obesity can impact negatively on mental health, why you still repeated same title, as well as tell the reader much more evidence to conduct the recent study, give the reasonable reason. Try to criticize the previous study.  The aim is clear. | Thank you for your comments and feedback. | Revised texts are in red color.  Page: 4 |
| Method section:  23% of the method section has been written by AI, please review it carefully and rewrite the sentences.  The authors wrote the study design and participants.  I recommend them to change the sub heading like (study setting, study design participants, questionnaire, and so on). | We are thankful to you for carefully checking the manuscript. We revised the Methods section. | Revised texts are in red color.  Page: 4-6 |
| Then please clarify why you select students only in one university, give the reason?. | Thanks for highlighting this point. This study is limited to a single university due to a lack of funding support; nevertheless, they intend to soon undertake a nationwide survey. We mention it in the Limitations section. | Revised texts are in red color.  Page: 14 |
| You reported that you used stratified sampling, same tell the reader why you select this method? And it is better to have and present the sampling technique through diagram. | Thanks. We revised the manuscript. | Revised texts are in red color.  Page: 4-6 |
| By which software you selected 600 students? Is it the total sample size? Or the whole number of the students? | Thanks. Actually, 600 students is the total sample size. We revised the manuscript. | Revised texts are in red color.  Page: 4-6 |
| You didn’t mentioned the exclusion criteria and inclusion criteria. You didn’t mention the consent was written paper or taken orally, please describe it. | Thanks. We add the inclusion and exclusion criteria. We now mentioned that we took oral consent from the respondents in the revised version of the manuscript. | Revised texts are in red color.  Page: 4-6 |
| Please mention the P value. | Thank you. We add it in the revised manuscript. | Revised texts are in red color.  Page: 6 |
| Result section: 22% has been written by AI, please change and paraphrase it. The whole section is good structured and well written. | Thanks for your inspirational comments. |  |
| Discussion section: 42% has been written by AI. You didn’t mention the strength points of the study please describe your strong points and then mention the limitation, during mentioning the limitations you didn’t use scientific terms such as report bias. You used some other words please review the words. Also, tell the redear how this type of bias can be effective on your result? | Thank you very much. We have revised the Discussion section. | Revised texts are in red color.  Page: 12-14 |
| Conclusion: 100% of discussion part has been by AI, please change the whole section and humanize it. And start with in conclusion or some other phrases which can be used for discussion part. Please don’t repeat the aim of the study inside conclusion part. | Thank you. We revised the Conclusion section. | Revised texts are in red color.  Page: 15 |
| In addition, add recommendations and the future work in the same area, as well as, your advise for students and the whole community. | Thanks. We add the recommendation and direction of further study at the end of the Conclusion section. | Revised texts are in red color.  Page: 15 |
| The reference list  The reference list has several issues, including incomplete citations, uneven formatting, missing DOIs or URLs, repetition, contradictory journal names and volume numbers, and inconsistent style. Some entries on the list lack DOIs or URLs, while others contain duplicate entries or cite comparable research more than once.  106 reference were used do you think all of them are necessary? Please revise them and use few of them. | Thanks. We revised the reference list. We add the citations using Mendeley software and follow the PLOS ONE style. | Revised texts are in red color.  Page: 16-20 |
| **Reviewer 2 Comments:** | **Response** | **Note** |
| Generally, the topic is good. The introduction is sound and has alluded to the research gap. However, the objectives mentioned at the end of the introduction is only part of what the author actually report in the results and discussion.  The method is too short. Need to elaborate more on the tools used including whether it is a validated questionnaire or if its newly developed, then the author need to explain how it was developed.  The results are more descriptive. In addition, it is not right for the author to claim association based on univariate analysis, hence it should not be presented and drawn conclusion based on it. | Thank you very much for your comments and feedback. We appreciate your comments. We revised the manuscript as per your comments and feedback.  The questionnaire which includes background information was developed based on the existing literature and self-efficacy. However, we also used some widely used and validated scales as we mentioned in the Methods section.  The association is identified by correlation and logistic regression model. | Revised texts are in red color.  Page: 1-15 |

Finally, the revised manuscript has been produced following the valuable comments and suggestions of the reviewers. Once again, we would like to thank the reviewers for their sincere dedication, professional insights, and earnest cooperation in reviewing the manuscript.
