# Supplementary material for: Prevalence of overweight and obesity and their impact on academic performance and psychological well-being among university students in 2024 in Bangladesh
Source: PLoS One. 2024 Dec 10;19(12):e0315321. doi: 10.1371/journal.pone.0315321 (PMC11630606; doi:10.1371/journal.pone.0315321)
Supplement: Attachment — Submitted filename: Response to Reviewers.docx [file pone.0315321.s007.docx]

**Author responses to the review comments:**

We would like to express our sincere gratitude to the reviewers and the Editors for their valuable comments. We have considered all the comments made by the reviewers and thoroughly revised and formatted the manuscript accordingly. A detailed response to each of the comments is provided below:

| **Academic Editor Comments:** | **Response** | **Note** |
| --- | --- | --- |
| Thank you for submitting your manuscript to PLOS ONE. After careful consideration, we feel that it has merit but does not fully meet PLOS ONE’s publication criteria as it currently stands. Therefore, we invite you to submit a revised version of the manuscript that addresses the points raised during the review process.  Please include the following items when submitting your revised manuscript:  A rebuttal letter that responds to each point raised by the academic editor and reviewer(s). You should upload this letter as a separate file labeled 'Response to Reviewers'.  A marked-up copy of your manuscript that highlights changes made to the original version. You should upload this as a separate file labeled 'Revised Manuscript with Track Changes'.  An unmarked version of your revised paper without tracked changes. You should upload this as a separate file labeled 'Manuscript'. | Thank you very much for carefully checking the manuscript and providing insightful comments.  All required files are uploaded to the journal system. | Revised texts are in red color. |
| **Journal Requirements:** | **Response** | **Note** |
| Please review your reference list to ensure that it is complete and correct. If you have cited papers that have been retracted, please include the rationale for doing so in the manuscript text, or remove these references and replace them with relevant current references. Any changes to the reference list should be mentioned in the rebuttal letter that accompanies your revised manuscript. If you need to cite a retracted article, indicate the article’s retracted status in the References list and also include a citation and full reference for the retraction notice. | Thanks. We checked the reference list and we confirm that they are correct. | Revised texts are in red color.  Page: 17-21 |
| **Additional Editor Comments:** | **Response** | **Note** |
| 1. Please remove the irrelevant citations such as 28, 29 and 30, and avoid of self citations. | Thank you again for a few additional comments. We removed the citations 28, 29, and 30. | Revised texts are in red color.  Page: 4 |
| 2. Please add another subheading in the method section regarding Validity and Reliability including Cronbach alpha values for all scales. | Thanks. We add the Cronbach alpha values for IPAQ-7 and RSES. | Revised texts are in red color.  Page: 6 |
| 3. The first paragraph of discussion should be your main results | Thanks. We revised the first paragraph of the Discussion section as per your comments. | Revised texts are in red color.  Page: 13 |
| 4. Add recommendation for future research | Thanks. In the final part of the conclusion section, we add future research directions. | Revised texts are in red color.  Page: 15 |

Finally, the revised manuscript has been produced following the valuable comments and suggestions of the reviewers. Once again, we would like to thank the reviewers for their sincere dedication, professional insights, and earnest cooperation in reviewing the manuscript.
